# The Antibiotic Resistance Profiles of Bacterial Strains Isolated from Patients with Hospital-Acquired Bloodstream and Urinary Tract Infections

**DOI:** 10.1155/2012/890797

**Published:** 2012-12-12

**Authors:** Hamed Ghadiri, Hamid Vaez, Samira Khosravi, Ebrahim Soleymani

**Affiliations:** ^1^Department of Microbiology, Faculty of Sciences, University of Tehran, Tehran 14155-6455, Iran; ^2^Department of Microbiology, School of Medicine, Isfahan University of Medical Sciences, P.O. Box 81748-84841, Isfahan 73461-8174, Iran; ^3^Department of Microbiology, Infection Control Committee, Besat Hospital, Tehran 14185-611, Iran

## Abstract

Treatment of nosocomial infections is becoming difficult due to the increasing trend of antibiotics resistance. Current knowledge on antibiotic resistance pattern is essential for appropriate therapy. We aimed to evaluate antibiotic resistance profiles in nosocomial bloodstream and urinary tract pathogens. A total of 129 blood stream and 300 urinary tract positive samples were obtained from patients referring to Besat hospital over a two-year period (2009 and 2010). Antibiotic sensitivity was ascertained using the Kirby-Bauer disk diffusion technique according to CLSI guidelines. Patient's data such as gender and age were recorded. The ratio of gram-negative to gram-positive bacteria in BSIs was 1.6 : 1. The most prevalent BSI pathogen was Coagulase-Negative *Staphylococci* (CoNS). The highest resistance rate of CoNS was against penicillin (91.1%) followed by ampicillin (75.6%), and the lowest rate was against vancomycin (4.4%). *Escherichia coli* was the most prevalent pathogen isolated from urinary tract infections (UTIs). Ratio of gram-negative to gram-positive bacteria was 3.2 : 1. The highest resistance rate of *E. coli* isolates was against nalidixic acid (57.7%). The present study showed that CoNS and *E. coli* are the most common causative agents of nosocomial BSIs and UTIs, and control of infection needs to be addressed in both antibiotic prescription and general hygiene.

## 1. Introduction

Nosocomial or hospital-acquired infections are defined as infections which are acquired during the hospital stay. Nosocomial infections are usually defined as infections that are identified at least 48–72 hours following admission to health institutions [1]. Nosocomial infections are also important public health problems in developing countries as well as in developed countries [2]. The most frequent types of nosocomial infections are urinary tract infection (UTI), surgical-wound infection, pneumonia, and bloodstream infection (BSI) [3]. BSIs are responsible for approximately 10–30% of the cases [4]. UTI is the presence of bacteria in the urine (bacteriuria) and defined as the growth of a single pathogen of >10^5^ colony-forming units/mL from properly collected mid-stream urine specimens [5]. The common bacterial pathogens present in the BSIs and UTIs are *Staphylococcus aureus, *Coagulase-Negative *Staphylococci* (CoNS), *Pseudomonas aeruginosa, Klebsiella pneumoniae, Escherichia coli, Enterobacter* spp., *Enterococcus* spp., and *Acinetobacter* spp. [6]. As the result of extensive uses of antimicrobial agents, nosocomial pathogens have shifted away from easily treatable bacteria towards more resistant bacteria. This change is important problem for nosocomial infection control and prevention [7]. Area-specific monitoring studies aimed to gain knowledge about type of pathogens and antimicrobial resistance patterns can optimize treatment and decrease mortality rates [8, 9]. In Iran, broad-spectrum antibiotics are commonly used in the hospitals, and there is limited data on antibiotic resistance. The present study was aimed to ascertain the resistance patterns of the most common bacterial isolates from hospital-acquired bloodstream and urinary tract infections over a two-year period.

## 2. Materials and Methods

### 2.1. Subjects

This study was conducted between January 2009 and December 2010 at Besat Hospital affiliated to Artesh University of Medical Sciences, Tehran, Iran. During the study, all patients admitted to different hospital wards with suspected nosocomial BSI or UTI were included. Patients who had been admitted for more than 48 h, with no sign of bacterial colonization at the time of admission were included. Clinical examination was conducted by physician to exclude community-acquired infections. Nosocomial UTI patients included in this study were classified in four age groups. This study was approved by the Ethics Committee of the Besat Hospital in Tehran.

### 2.2. Sample Collection and Bacterial Identification

For BSI, an appropriate volume of blood sample was collected with aseptic technique. Two independent blood samples for a patient were recruited. Obtained samples were inoculated into Castaneda (biphasic blood culture bottles) medium, immediately and incubated in aerobic conditions with and without CO_2_. Primary check was done after 24 hours. Constitutive check was performed until 21 days. Subculture on ship blood, chocolate, and Mac Conkey agar plates was performed for suspected Castaneda inoculated specimens [10, 11]. Multimicrobial growth on culture media was excluded. Nonrepetitive positive cultures were recruited for study. In UTI suspected patients, the midstream specimens of urine were taken following the recommendations of Kass [12]. The plates were incubated in aerobic atmosphere at 37°C for 24–48 hrs. Presence of more than 10^5^ (cfu/mL) bacteria signified as UTI. Isolated bacteria were microbiologically identified with standard biochemical identification methods [13, 14].

### 2.3. Antibiotic Susceptibility Testing

Antibiotic susceptibility testing was performed by Kirby-Bauer's disk diffusion method on Muller-Hinton agar (Hi Media, Mumbai, India) in accordance with the standards of the Clinical Laboratory Standards Institute (CLSI, formerly National Committee for Clinical Laboratory Standards [NCCLS]) guidelines [15]. The antibiotic concentration per disk was as follows: penicillin 10 units, ampicillin 10 *μ*g, erythromycin 5 *μ*g, ceftazidime 30 *μ*g, ciprofloxacin 5 *μ*g, cotrimoxazole 25 *μ*g, gentamycin 10 *μ*g, tetracycline 10 *μ*g, amikacin 30 *μ*g, imipenem 10 *μ*g, nitrofurantoin 30 *μ*g, nalidixic acid 30 *μ*g, clindamycin 2 *μ*g, oxacillin 1 *μ*g, cefoxitin 10 *μ*g, cephalothin 30 *μ*g, and vancomycin 30 *μ*g. *S. aureus* ATCC 25923 and *E. coli* ATCC 25922 were used as control strains.

### 2.4. Statistical Analysis

Data entry and statistical analysis were performed using SPSS v.13 software. Comparisons were made using Pearson Chi-square and Fisher exact tests. A *P* value of <0.05 was considered indicative of a statistically significant difference.

## 3. Results

### 3.1. Nosocomial BSI

A total of 1200 blood samples which were obtained from nosocomial BSI of suspected patients were analyzed. Among them, 129 (10.7%) were documented BSI with monomicrobial origin. Of the 129 patients, 61 (47.3%) were females and 68 (52.7%) were males (*P* > 0.05). 

**Figure 1:**
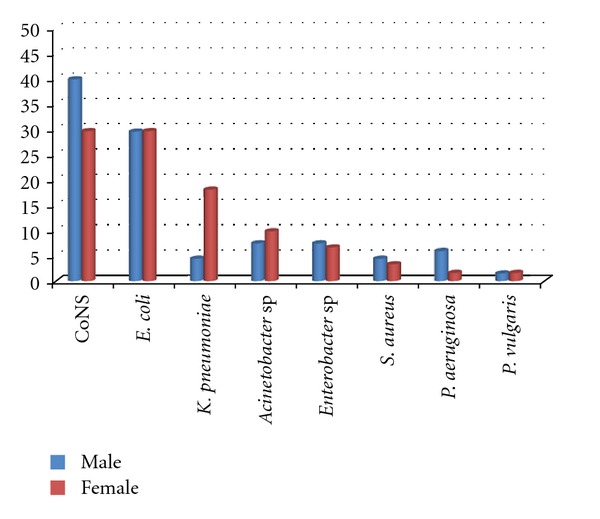
Frequency and distribution of bacterial isolates from blood cultures. CoNS*: Coagulase-Negative *Staphylococci. *

CoNS (34.8%) and *E. coli* (29.4%) were the most prevalent microorganisms isolated from nosocomial BSI patients followed by* K. pneumoniae* (11%), *Acinetobacter* spp. (8.5%), *Enterobacter* spp. (7%), *S. aureus* (3.9%), *P. aeruginosa* (3.9%), and *Proteus vulgaris* (1.5%).  Figure 1 represents the gender wise prevalence of bacteria isolated from blood cultures. No significant correlation was observed between gender and BSIs (*P* > 0.05). The highest resistance rate of the CoNS was against penicillin (91.1%) followed by ampicillin (75.6%), and the lowest rate was against vancomycin (4.4%). *Proteus vulgaris* was the least prevalent bacteria isolated from blood cultures and had a high sensitivity to different antibiotics (data not shown). *E. coli* had the highest resistance rates to tetracycline (63.2%) and ampicillin (63.2%), while imipenem and gentamycin showed the lowest level of resistance (15.8%). The resistance rates of bacteria isolated from nosocomial BSI against different antibiotics are presented in Table 1.

### 3.2. Nosocomial UTI

A total of 1000 urine samples which were obtained from patients suspected of nosocomial UTI were analyzed. Among them, 300 samples (30%) were distinguished as UTIs, which were isolated from 204 (68%) females and 96 (32%) males (*P* < 0.05), respectively (mean age: 46.8 years, range: 15–91 years). The most frequency of UTIs (22.7%) was found in females with mean age of >60 yrs (Table 3). *E. coli* (66.7%) was the most common gram-negative bacilli isolated from nosocomial UTI patients followed by CoNS (11.7%), *S. aureus* (6.7%), *Proteus vulgaris* (6.7%), *Enterococcus* spp. (5%), *P. aeruginosa* (1.7%), and *Enterobacter* spp. (1.7%). The highest resistance rate of *E. coli* was against nalidixic acid (57.7%). Results of the resistance rates to different antibiotics were showed in Table 2.

## 4. Discussion

Nosocomial infections occur worldwide and affect both developed and developing countries [16]. Many of these infections are associated with microorganisms that are resistant to antibiotics and can easily spread by hospital personnel [17]. Guidelines for antibiotic therapy can be helpful for clinicians to select more appropriate antibiotics for effective treatment and prevent the development of drug resistance. This study shows the distribution of antibiotic resistance pattern of bacterial species isolated from patients with nosocomial BSI or UTI at a hospital in Tehran, Iran.

This study revealed that 129 (10.7%) out of 1200 bloodstream samples which were obtained from nosocomial BSI suspected patients were positive. Of the 129 patients, 61 (47.3%) were females and 68 (52.7%) were males (*P* > 0.05). The CoNS (34.8%) and *E. coli* (29.4%) were the most prevalent microorganisms that have been isolated. Similar findings have been observed in Turkey and in a Children's Medical Center, Iran [18, 19]. A study in Brazil revealed the predominance of *S. aureus* (14%) followed by CoNS (12.6%) and *Klebsiella* (12%) [20]. In several studies, CoNS followed by *S. aureus* comprised the most prevalent bacteria isolated from BSIs [21–23]. In our study, gram-negative bacteria were more regularly involved in nosocomial BSI than gram-positive bacteria (*P* < 0.05). This finding is in accordance with the results of recent studies [24]. Based on our data, the highest resistance rate of the CoNS was against penicillin followed by ampicillin and oxacillin (Table 1). Oxacillin-resistant *staphylococcus* spp. are an increasing global problem in nosocomial infections [25–27]. Oxacillin-resistant strains show the high level of resistance to penicillin, cephalosporins, and other beta-lactams like imipenem. In the present study, 62% of the isolated CoNS were oxacillin resistant with the high rates of resistance to penicillin, cephalothin, and imipenem (100%, 100%, and 62%, resp.). *E. coli* strains which were isolated from BSI patients had the highest resistance rates to tetracycline and ampicillin (63.2%), which is similar to the recent study from Ireland [28]. In the present study, the *E. coli* strains which were isolated from BSI patients showed a resistance rate of 47.4% to ciprofloxacin which is consistent with the other studies from Iran [29, 30]. In our study, the 8.5% of microorganisms which were isolated from nosocomial BSI patients were *Acinetobacter* spp. The *Acinetobacter* spp. isolates showed the highest resistance rate to cephalothin (81.8%) followed by cotrimoxazole and gentamicin (63.6%) (Table 1). Reports of *Acinetobacter* spp. bacteremia are increasing, especially from Asian countries and neighboring countries of Iran such as Iraq, Kuwait, Turkey, and Afghanistan [31–33]. A recent surveillance study from Iran reported that *Acinetobacter* spp. were the most frequently isolated bacteria in the hospital and community-acquired BSIs (32%) followed by *E. coli* (13.7%) and *Klebsiella* sp. (12%), respectively [28]. In the present study, vancomycin was the most effective antibiotic against CoNS (95.6% susceptible) and *S. aureus* (100% susceptible). This is in agreement with another study performed in Iran [34]. In our study, statistical analysis showed a significant correlation between nosocomial UTI prevalence and gender (*P* < 0.05). It has been extensively reported that adult women have a higher prevalence of UTI than men because of anatomic and physical situations [35, 36]. 

The present study indicates that *E. coli* is still the most common cause of nosocomial UTI. This finding is consistent with the other studies from Iran and other countries [35, 37, 38]. The highest resistance rate of *E. coli* isolate which was obtained from urine samples was against nalidixic acid followed by cotrimoxazole, ciprofloxacin, and ampicillin, respectively (Table 2). These results were predictable because these antibiotics have been used as a long time in our hospital. In this study, amikacin and imipenem had the widest coverage against *E. coli* isolates (97.5%). In a recent surveillance study in Iran authors reported that the highest resistance rate of *E. coli* isolates which were obtained from various clinical specimens at 11 hospitals was against tetracycline followed by amoxicillin and penicillin, respectively [39].

The rate of nosocomial UTI is determined by the interactions of several factors such as primary disease and its severity, duration of hospitalization and treatment, and invasive interventions like use of urinary catheters; Moreover, the incidence of nosocomial UTI has been increasing and its treatment has become more complicated because of the pathogens with increasing resistance to antibiotics [40, 41].

## 5. Conclusion

It is concluded that in our hospital gram-negative bacteria were more frequently involved in nosocomial BSI than gram-positive bacteria. CoNS and *E. coli* were the most common isolated bacteria from blood cultures and included the 64% of total isolates. Vancomycin was the most effective antibiotic against gram-positive bacteria, and gentamicin, amikacin, and imipenem are proposed for treatment of nosocomial UTI caused by Gram-negative bacteria. *E. coli* was the most common cause of nosocomial UTI in our hospital and imipenem and aminoglycosides (gentamicin, amikacin) were the most effective antibiotics against this infection. Finally, to reduce the incidence of nosocomial infections, the appropriate use of antibiotics according to the standard antimicrobial susceptibility tests is proposed.

## Figures and Tables

**Table 1:** Antimicrobial resistance profiles of hospital-acquired bacterial isolates from clinical blood specimens.

Antimicrobial agents	Microorganisms							Total *n* (%)
	CoNS* *n* (%)	*E*. *coli* *n* (%)	*K*. *pneumonia* *n* (%)	*Aceinetobacter * spp.*n* (%)	*En* *te* *ro* *ba* *c* *te* *r* spp. *n* (%)	*S*. *aureus* *n* (%)	*P*. *aeruginosa* *n* (%)	
Ciprofloxacin	22 (48.9)	18 (47.4)	6 (42.9)	5 (45.5)	1 (11.1)	2 (40)	2 (40)	56 (43.4)
Ceftazidime	18 (40)	15 (39.5)	6 (42.9)	6 (54.5)	3 (33.3)	3 (60)	3 (60)	54 (41.9)
Amikacin	20 (44.4)	6 (15.8)	6 (42.9)	6 (54.5)	0 (0)	3 (60)	3 (60)	44 (34.1)
Imipenem	16 (35.6)	6 (15.8)	6 (42.9)	6 (54.5)	2 (22.2)	4 (80)	4 (80)	44 (34.1)
Clindamycin	28 (62.2)	19 (50)	6 (42.9)	6 (54.5)	2 (22.2)	3 (60)	4 (80)	68 (52.7)
Vancomycin	2 (4.4)	NA*	NA	NA	NA	0 (0)	NA	2 (1.6)
Cephalothin	22 (48.9)	16 (42.1)	9 (64.3)	9 (81.8)	3 (33.3)	2 (40)	3 (60)	64 (49.6)
Ampicillin	34 (75.6)	24 (63.2)	7 (50)	7 (63.6)	4 (44.4)	5 (100)	5 (100)	86 (66.7)
Cotrimoxazole	26 (57.8)	19 (50)	12 (85.7)	7 (63.6)	1 (11.1)	5 (100)	5 (100)	75 (58.1)
Cefoxitin	26 (57.8)	17 (44.7)	8 (47.1)	6 (54.5)	2 (22.2)	4 (80)	4 (80)	67 (51.9)
Oxacillin	28 (62.2)	NA	NA	NA	NA	2 (40)	NA	30 (23.3)
Erythromycin	17 (37.8)	13 (34.2)	6 (42.9)	4 (36.4)	1 (11.1)	3 (60)	4 (80)	48 (37.2)
Tetracycline	23 (51.1)	24 (63.2)	9 (64.3)	5 (45.5)	6 (66.6)	4 (80)	5 (100)	76 (58.9)
Penicillin	41 (91.1)	22 (58)	11 (78.6)	7 (63.6)	4 (44.4)	5 (100)	5 (100)	95 (73.6)
Gentamicin	16 (35.6)	6 (15.8)	4 (28.6)	7 (63.6)	2 (22.2)	4 (80)	2 (40)	41 (31.8)

Total	45 (34.8)	38 (29.4)	14 (11)	11 (8.5)	9 (7)	5 (3.9)	5 (3.9)	127 (98.5)

CoNS*: Coagulase-negative *Staphylococci*.

NA*: Not applicable.

**Table 2:** Antimicrobial resistance profiles of hospital-acquired bacterial isolates from clinical urine specimens.

Antimicrobial agents	Microorganisms							Total *n* (%)
	CoNS* *n* (%)	*E*. *coli* *n* (%)	*P*. *vulgaris* *n* (%)	*Enterococcus* sp. *n* (%)	*En* *te* *ro* *ba* *c* *te* *r* sp. *n* (%)	*S*. *aureus* *n* (%)	*P*. *aeruginosa* *n* (%)	
Ciprofloxacin	20 (57.1)	80 (40)	10 (50)	7 (46.6)	2 (40)	3 (15)	3 (60)	125 (41.6)
Ceftazidime	15 (42.8)	18 (9)	8 (40)	4 (26.6)	1 (20)	4 (20)	3 (60)	53 (17.7)
Amikacin	22 (62.8)	5 (2.5)	7 (35)	6 (40)	1 (20)	5 (25)	3 (60)	49 (16.3)
Imipenem	10 (28.5)	5 (2.5)	4 (20)	2 (13.3)	0 (0)	5 (25)	2 (40)	28 (9.3)
Clindamycin	20 (57.1)	19 (9.5)	4 (20)	8 (53.3)	1 (20)	6 (30)	3 (60)	61 (20.3)
Nalidixic acid	7 (20)	115 (57.7)	7 (35)	7 (46.6)	1 (20)	6 (30)	4 (80)	147 (49)
Cephalothin	18 (51.4)	20 (10)	8 (40)	10 (66.6)	1 (20)	6 (30)	4 (80)	67 (22.3)
Ampicillin	34 (97.1)	80 (40)	14 (70)	12 (80)	2 (40)	18 (90)	5 (100)	165 (55)
Cotrimoxazole	20 (57.1)	100 (50)	10 (50)	10 (66.6)	1 (20)	10 (50)	3 (60)	154 (51.3)
Nitrofurantoin	28 (80)	80 (40)	13 (65)	7 (46.6)	2 (40)	8 (40)	3 (60)	141 (47)
Erythromycin	15 (42.8)	43 (21.5)	7 (35)	4 (26.6)	1 (20)	12 (60)	2 (40)	84 (28)
Tetracycline	25 (71.4)	24 (12)	3 (15)	5 (33.3)	1 (20)	10 (50)	3 (60)	71 (23.7)
Penicillin	35 (100)	55 (27.5)	12 (60)	13 (86.6)	3 (60)	18 (90)	5 (100)	141 (47)
Gentamicin	10 (28.5)	10 (5)	6 (30)	9 (60)	2 (40)	9 (45)	2 (40)	48 (16)

Total	35 (11.7)	200 (66.7)	20 (6.7)	15 (5)	5 (1.7)	20 (6.7)	5 (1.7)	300 (100)

CoNS*: Coagulase-negative Staphylococci.

**Table 3:** Age and gender distribution in hospital-acquired UTI patients.

Gender	Age groups				Total *n *(%)
	<20 *n* (%)	20–40 *n* (%)	40–60 *n* (%)	>60 *n* (%)	
Male	15 (5)	24 (8)	26 (8.6)	31 (10.3)	96 (32)
Female	30 (10)	51 (17)	55 (18.3)	68 (22.7)	204 (68)

Total	45 (15)	75 (25)	81 (26.9)	99 (33)	300 (100)
